# An ultrasound-absorbing inflorescence zone enhances echo-acoustic contrast of bat-pollinated cactus flowers

**DOI:** 10.1242/jeb.245263

**Published:** 2023-03-03

**Authors:** Ralph Simon, Felix Matt, Vinicio Santillán, Marco Tschapka, Merlin Tuttle, Wouter Halfwerk

**Affiliations:** ^1^ Department of Ecological Science, Vrije Universiteit, 1081 HV Amsterdam, The Netherlands; ^2^ Behavioral Ecology and Conservation Lab, Nuremberg Zoo, 90480 Nuremberg, Germany; ^3^ Cosys-Lab, Faculty of Applied Engineering, University of Antwerp, 2020 Antwerpen, Belgium; ^4^ Estación Cientóf́ica San Francisco, Loja, Ecuador; ^5^ Faculty of Geography, Lab for Climatology and Remote Sensing, Philipps-University of Marburg, 35032 Marburg, Germany; ^6^ Unidad Académica de Posgrado, Universidad Católica de Cuenca, Cuenca 010107, Ecuador; ^7^ Institute of Evolutionary Ecology and Conservation Genomics, University of Ulm, 89069 Ulm, Germany; ^8^ Smithsonian Tropical Research Institute, Ancon, Balboa, Panama; ^9^ Merlin Tuttle’s Bat Conservation, MerlinTuttle.org, Austin, TX 78735, USA

**Keywords:** Floral acoustics, Echolocation, Bat pollination, Chiropterophily, Cactaceae, Cephalium, Ultrasound absorption

## Abstract

Flowering plants have evolved an extraordinary variety of signalling traits to attract their pollinators. Most flowers rely on visual and chemical signals, but some bat-pollinated plants have evolved passive acoustic floral signals. All known acoustic flower signals rely on the same principle of increased sonar reflectivity. Here, we describe a novel mechanism that relies on increased absorption of the area surrounding the flower. In a bat-pollinated cactus (*Espostoa frutescens*) we found a hairy inflorescence zone, a so-called cephalium. Flowers solely emerge out of this zone. We measured the echoes of cephalia, flowers and unspecialized column surfaces and recorded echolocation calls of approaching bats. We found that the cephalium acts as a strong ultrasound absorber, attenuating the sound by −14 dB. The absorption was highest around the echolocation call frequencies of approaching bats. Our results indicate that, instead of making flowers more reflective, plants can also evolve structures to attenuate the background echo, thereby enhancing the acoustic contrast with the target.

## Teaser

**Highlighted Article:** An Ecuadorian cactus acoustically advertises its flowers to bats with a sound-absorbing structure. Sound dampening is achieved by fine hairs on the surface of the cactus.

## INTRODUCTION

Flowering plants rely on a wide variety of communication strategies to attract their pollinators. Conspicuous visual flower signals in particular are useful to guide receivers, as they are easy to locate and the use of colours makes flowers stand out against the vegetation background (Spaethe et al., 2001; Forrest and Thomson, 2009; Trunschke et al., 2021). Neotropical bat-pollinated plants are limited in the use of visual signals to attract their pollinators and therefore independently evolved acoustic traits (Simon et al., 2021) or even echo-reflective structures to acoustically guide these nocturnal pollinators (von Helversen and von Helversen, 1999; von Helversen et al., 2003; Simon et al., 2011). Echo-acoustic signalling plants all use concave shapes with triple mirror, bell-like or dish-like structures. These concave structures share the same basic acoustic principle of focusing returning echoes to an approaching bat, thereby increasing the range over which they can be detected. Some flower signals use additional spectral–temporal signatures to increase conspicuousness (Simon et al., 2011). These echo signatures are generated by interference between sound pathways on the reflector surface that causes enhancement of certain frequency bands (Simon et al., 2020). Reflective plant structures evolved not only in the Neotropics but also in a bat-dependent pitcher plant from Borneo, *Nepenthes hemsleyana*. This pitcher plant depends on bats roosting inside the pitcher as they provide additional nitrogen through their droppings (Grafe et al., 2011). These plants therefore developed a reflective prolonged pitcher backwall to advertise their pitcher leaves as roosts (Schöner et al., 2015). The fact that reflective plant structures independently evolved several times in different ecological contexts and in different plant families shows how important such signals are for ecological networks.

Here, we assessed a novel evolutionary adaptation that enhances acoustic communication between plants and pollinating bats. Some cactus species exhibit inflorescence zones on their column that are particularly hairy, the so-called cephalium (see Fig. S1). There are several different morphologies of cacti that are termed cephalia, and we refer here to what is described as a lateral cephalium by Mauseth (2006). Several functions of these cephalia zones have been proposed. The hairy structure may shield buds from UV radiation at high altitudes, or protect against nectar robbers and herbivores (Buxbaum, 1961; Martorell et al., 2006; Mauseth, 2006). We tested a hypothesis by von Helversen et al. (2003), which states that such hairy zones may have been co-opted to serve in bat-pollinated cacti as sound-absorbing structures that support detection and localization of sound-reflecting flowers by pollinating bats.

Using a bat-mimetic sonar-head, we carried out ensonification experiments with different structures of the cactus *Espostoa frutescens* (von Helversen et al., 2003) from the Andes*.* Specifically, we ensonified the cactus' column, flowers as well as the hairy cephalium zone. Additionally, we recorded the echolocation calls of its main pollinator, the nectar-feeding bat *Anoura geoffroyi* (Phyllostomidae), and assessed whether the cephalium was especially absorbent in the ultrasonic frequency range of the calls.

## MATERIALS AND METHODS

We studied *Espostoa frutescens* Madsen and its pollinator, Geoffroy's tailless bat (*Anoura geoffroyi* Gray 1838). The study was carried out in a dry valley of the Ecuadorian Andes, close to the city of Oña in the province of Azuay. As it was not possible to conduct the echo measurements in the field – the cacti grow in rocky and steep habitats – we had to cut some columns (*n*=6) and conducted the measurements indoors at a nearby farm. All experiments were approved by the local authorities (Ministeria del Ambiente, Cuenca, Ecuador, autorizacion para investigación científica no. 035-DPA-MA-2012). A specimen of *E. frutescens* is deposited at Herbario Azuay (Cuenca, Ecuador) with the number HA 7814.

To measure the reflectance of the different structures of the cacti, we mounted the columns on tripods and used a custom-built biomimetic sonar head to ensonify them. The sonar-head consisted of a 1/4 inch condenser microphone (40BF; preamplifier 26AB; power module 12AA; G.R.A.S. Sound & Vibration, Holte, Denmark) and a custom-made EMFi (Electro Mechanical Film) loudspeaker (mean±s.d. sound pressure level at 1 m distance: 92±8 dB, frequency range: 30–160 kHz; Department of Sensor Technology, University of Erlangen-Nuremberg, Erlangen, Germany). The speaker and the microphone were embedded in an aluminium body and were placed next to each other, similar to how the mouth/nose and ears are arranged on a bat's head. To ensure a quite narrow sound beam similar to that of a nectar-feeding bat, we ensonified cacti from a relatively short distance of 15 cm. As we measured the impulse responses (echo responses of very short pulses), we had no problem with overlap. We obtained the impulses by ensonifying with a continuously replayed MLS (maximum length sequence) signal and recorded the reflected sound. MLS is a periodic pseudorandom binary sequence, basically a predetermined noise signal and a basic property of any MLS signal is that their autocorrelation function is perfectly narrow; therefore, it is possible to obtain the impulse responses (IRs) by deconvolution of the reflected echo and the original MLS (von Helversen et al., 2003). The advantage of ensonifying with an MLS instead of any bat-like sweep or short impulse is that it is possible to ensonify with more sound energy and therefore the obtained echoes have a much better signal-to-noise ratio. The spectra of the echoes were obtained by windowing the IRs (rectangular, 1024 samples) around the echo of the cactus and then calculating the power spectral density (PSD). To obtain spectral target strength, independent of the frequency response of the loudspeaker, we conducted another measurement in which the cactus was replaced by a plate made from acrylic glass, which was oriented perpendicular to the direction of sound propagation. We deduced the PSD of the total reflection of that acrylic plate and then calculated the difference between the PSD from the recordings of the cactus surfaces and the PSD of the ‘total reflection’ of the plate (for more information on the setup and the signal processing, see von Helversen et al., 2003; Simon et al., 2006, 2011).

Using our ensonification setup, we measured the acoustic properties of six freshly cut columns of *E. frutescens*, focusing on the hairy cephalium zone and the unspecialized parts (backside) of the column. For both measurements, we scanned the columns by moving the sonar-head upwards along its vertical axis and made 10 measurements at different heights of the column. We also measured the reflectance of six isolated flowers, which were mounted on a stepping motor. We rotated the flower in 6 deg steps and measured 10 echoes around the opening (0 deg) of the flowers from −30 to 30 deg.

To deduce an average target strength for each structure and each cactus, we averaged the target strength for the 10 recordings we had of every structure (see Fig. S2). We also averaged target strength for five different frequency bands. One frequency band covered the whole bandwidth that we could measure with our setup. The other frequency bands were chosen to split the whole bandwidth in four logarithmically spaced frequency bands. We did this because auditory perception shows an exponential frequency distribution along the cochlea (for more information on spectral echo perception, see Simon et al., 2006).

To understand how the echo of a flower would be received if *E. frutescens* had no cephalium structures, we manipulated one column and mimicked a flower growing on an unspecialized part of the column. We first scanned the hairy cephalium with an open flower by moving the sonar-head upwards along the vertical axis of the column over an area of 30 cm. The flower was located centrally within this area and we measured in 1 cm steps. After the measurements, we resected the flower from the cephalium and fixed it on the hairless back of the column (Fig. 2B). For this experimentally manipulated column, we made the same detailed vertical scan (30 cm, 1 cm steps).

We also recorded echolocation calls of two male Geoffroy's tailless bats (*A. geoffroyi*) approaching an *E. frutescens* column with an open flower. The microphone (1/4 inch condenser microphone 40BF; preamplifier 26AB; power module 12AA; G.R.A.S. Sound & Vibration) was placed next to the flower (see Fig. S3) and we recorded with a sampling rate of 500 kilo samples s^−1^. We obtained 45 manually triggered recordings, each with a length of 2 s, during the approaches of the bats. To ensure a good signal-to-noise ratio for the call analysis, we selected 21 approach sequences where at least two calls had a maximum amplitude of more than 60 mV. We analysed the calls using the program Avisoft-SASLab Pro (Avisoft Bioacoustics, Glienicke, Germany).

We tested for significant effects of plant structure on echo-acoustic target strength using the lmer package in R (version 3.5.3). We constructed linear mixed models (LMMs) and checked model assumptions by visual inspection of the residuals. Target strength was averaged over the 10 measurements per plant individual and structure and modelled as a dependent variable. Plant structure (column, flower or cephalium) was added as a fixed factor and plant individual as a random intercept term. For the different frequency ranges, we modelled the interaction between plant part and frequency band. We tested for significance of the main effect of plant structure on target strength and for significance of the interaction between structure and frequency band by comparing models with and without terms using likelihood ratio tests.

## RESULTS AND DISCUSSION

**Fig. 1.**
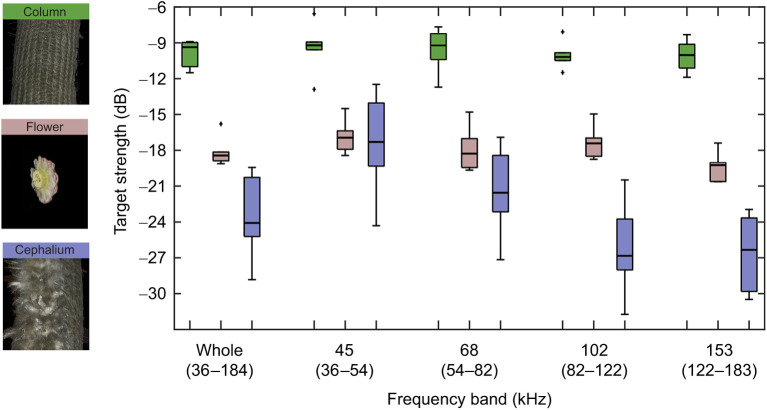
**Spectral target strength of different morphological structures of *Espostoa frutescens* for different frequency bands.** The spectral target strength was obtained from ensonification measurements at a distance of 15 cm. We measured unspecialized parts of the cactus column (green; *n*=6 columns, 10 measurements per column), isolated flowers (pink; *n*=6 columns, 10 measurements per flower from different angles) and the hairy cephalium zone (purple; *n*=6 columns, 10 measurements per column). Lines within boxplots are the median and whiskers show the non-outlier minimum and maximum.

We found a significant effect of plant structure on overall target strength (LMM, *n*=18 plant structures, *n*=180 measurements, d.f.=2, χ^2^=39.31, *P*<0.001). Furthermore, target strength depended on the interaction between frequency range and plant structure (LMM, d.f.=8, χ^2^=37.51, *P*<0.001). Overall, the plain column surface of *E. frutescens* reflected the strongest echoes. We measured a high target strength (average −9.8 dB) for these unspecialized surfaces of the cactus across a wide range of frequencies (Fig. 1). The overall average target strength of the flower was much lower than that of the column (−18.1 dB) but also remained similar across all measured frequency bands (Fig. 1). The hairy cephalium zone, in contrast, showed differences in target strength for the different frequency bands (Fig. 1). For the lower frequency band (45 kHz), the target strength was about the same level as that of the flower (−17.5 dB) but for higher frequency bands it was much lower, down to −26.3 dB for the 102 kHz frequency band. Overall, the cephalium zone had an average target strength of −23.7 dB, which is around 14 dB lower than that of the unspecialized parts of the column. The averaged full spectra (Fig. S2) of the different structures showed the same pattern. Flower spectra and cephalium spectra start to diverge from 70 kHz up to higher frequencies, with a maximum at around 150 kHz, where they had an average difference of over 10 dB.

**Fig. 2.**
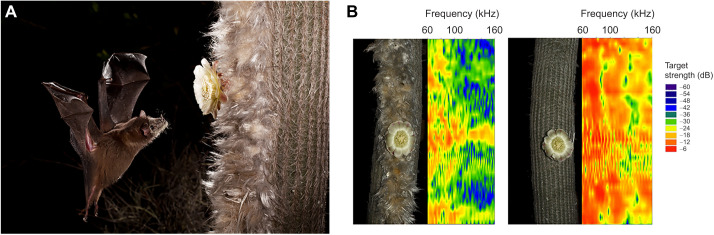
**A nectar-feeding bat approaching a flower and echo fingerprints of different cactus surfaces with flowers.** (A) Image of a Geoffroy's tailless bat (*Anoura geoffroyi*) approaching a flower of *E. frutescens*, which is embedded in the hairy cephalium zone (photo credit: ©MerlinTuttle.org). (B) Echo fingerprints of acoustic scans along the cactus column. On the left is a natural column with cephalium and flower; on the right is an experimentally manipulated column. The flower was cut out of the hairy zone and fixed on an unspecialized part of the column. The intensity (spectral target strength in dB) of the echo is given in colour gradation (red indicates high intensity, blue low intensity).

A qualitative analysis of the echo-acoustic fingerprint of specialized versus unspecialized parts of the column provided more detailed insight into the effect of the background on the detectability of flower targets (Fig. 2B). The unspecialized column reflects high target strength echoes for almost the entire bandwidth, which only in some areas are interrupted by some frequency notches. The specialized cephalium side of the column reflects much less sound energy, especially for frequencies above 90 kHz. When scanning the column at the position of the flower, the unmanipulated flower stands out from the less-reflecting background, in particular at frequencies above 90 kHz. When we placed a flower on the unspecialized part of the column, the flower echoes almost completely disappeared within the loud background echoes, although there might have been some additional interference patterns affecting the target strength.

**Fig. 3.**
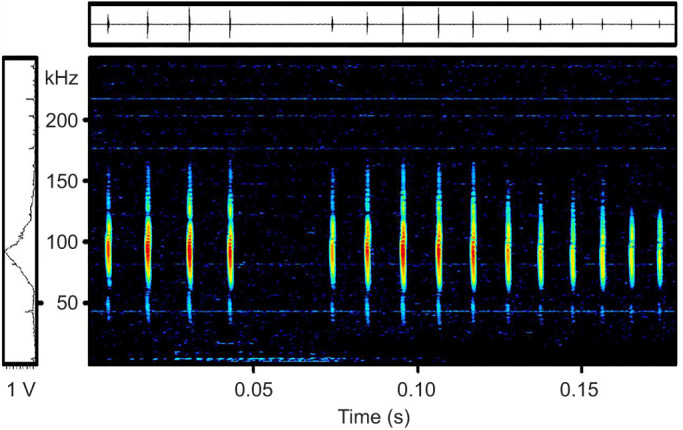
**A typical series of calls of a Geoffroy's tailless bat approaching an *E. frutescens* flower.** The microphone was placed next to the flower, as shown in Fig. S3.

In total we analysed 279 echolocation calls of two individuals of *A. geoffroyi* (see Fig. 3 for an example of an echolocation call sequence during an approach to an *E. frutescens* flower). The calls were short, having a duration of only 0.47±0.18 ms (mean±s.d.) and they were step frequency modulated starting at 132.7±8.1 kHz and ending at 59.8±10.0 kHz. The peak frequency of the calls was 92.5±4.4 kHz, which falls into the frequency band where sound absorption of the cephalium was highest.

Our ensonification experiments revealed distinct and frequency-dependent differences in echo-acoustic reflectance of different cactus parts. We found that the plain column of *E. frutescens* acts as a strong reflecting surface as it is cylindrical, providing reflective surfaces in all directions, and also because the surface has ridges, which may additionally act as small retroreflectors. In the right spectra of Fig. 2B there are frequency bands that cover 30–40 kHz. These bands could be caused by frequency interference resulting from the ridges, which are about 0.5 cm deep (see Fig. S1B). The flowers of *E. frutescens* reflect much less energy compared with the column, mainly because the reflecting surface is smaller and the flowers have a lot of anthers that scatter sound energy. The specialized cephalium surrounding the flowers reflected the least energy, in particular in the echolocation call frequency range of the plant's main pollinator, *A. geoffroyi.* These results strongly suggest that the cephalium of *E. frutescens* functions as a sound-absorbing structure and thus enhances the echo-acoustic contrast between the flower and the vegetative part of the plant for an approaching bat. While scanning cactus columns for flowers along the cephalium, the bats will receive faint echoes unless their call hits a flower, which increases the echo response by around 10 dB. In contrast, flowers growing on the unspecialized parts of the column would be much more difficult to detect in front of the highly reflective background. Bats might be able to perceive the interference patterns caused by the flowers; however, this would require much more processing than a salient flower echo in front of an absorbing surface.

As nectar-feeding bats have to visit or revisit several hundred flowers each night to cover their nightly energy expenditure (Winter and von Helversen, 2005), this simple yet efficient mechanism of dampening the background of the flowers may help the bats to save on foraging time and thus increase foraging efficiency. In turn, the plant will benefit from a higher cross-pollination rate. Bats are very efficient pollinators that carry a lot of pollen in their fur (see Fig. 2A) and have a huge home range so they can pollinate plants growing far apart (von Helversen and Winter, 2003).

The absorption of the cephalium is most efficient for the 102 kHz frequency band (82–122 kHz), which translates to a wavelength of around 3.4 mm (4.2–2.8 mm). The microstructure of the cephalium apparently favours absorption of sound around this wavelength, while larger wavelengths (e.g. 7.6 mm for the 45 kHz band) are less attenuated (difference of around 10 dB). The hairs are much smaller in diameter than the wavelengths of sound they absorb best and therefore probably do not scatter the incoming sound waves. An alternative explanation could be that the hairs create a layer of air with different temperature and humidity that reflects the sounds in a frequency-dependent manner.

As other species of *Espostoa* show similar hairy inflorescence zones, this floral acoustic adaptation might not be limited to this species alone. Hairy cephalia zones are found, for example, in the genus *Microanthocereus* (Mauseth, 2006). Interestingly, bird-pollinated species of the genus *Microanthocereus* also have cephalium zones; however, the fur is less dense (R.S. and W.H., personal observation). We argue that cephalium-like structures originally evolved for protection of floral structures, but were co-opted at some point in time to serve an additional or new functional role in pollinator attraction. Once co-opted, the cephalium of bat-pollinated flowers became optimized for this new function through selection by the echolocating bat pollinators.

Our study shows that bat-pollinated flowers can rely on absorption in addition to reflectance as an acoustic adaptation towards their pollinators. Echoacoustic absorption probably plays a much larger role across a wide range of ecological contexts (for example, fruit dispersal context, predator–prey context) than has so far been appreciated. Sound-absorbent structures have already been described for moth scales (Shen et al., 2018) as well as for thoracic moth fur (Neil et al., 2018). However, whether absorption has adapted in the context of predator–prey arms races remains to be tested, ideally in a comparative phylogenetic framework.

## Supplementary Material

10.1242/jexbio.245263_sup1 Supplementary information
