# Supplementary material for: An ultrasound-absorbing inflorescence zone enhances echo-acoustic contrast of bat-pollinated cactus flowers
Source: J Exp Biol. 2023 Mar 3;226(5):jeb245263. doi: 10.1242/jeb.245263 (PMC10038143; doi:10.1242/jeb.245263)
Supplement: Supplementary information [file jexbio-226-245263-s1.pdf]

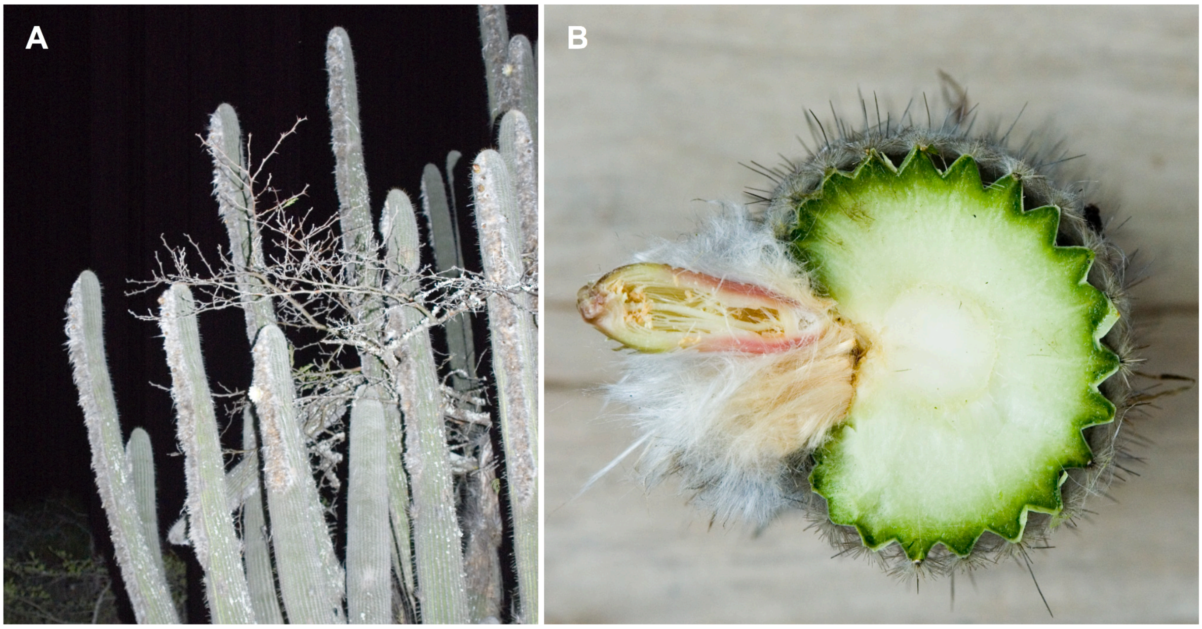

Fig. S1. (**A**) Habitus of an *Espostoa frutescens* plant and (**B**) cross section of a column with the hairy cephalium and a closed flower.

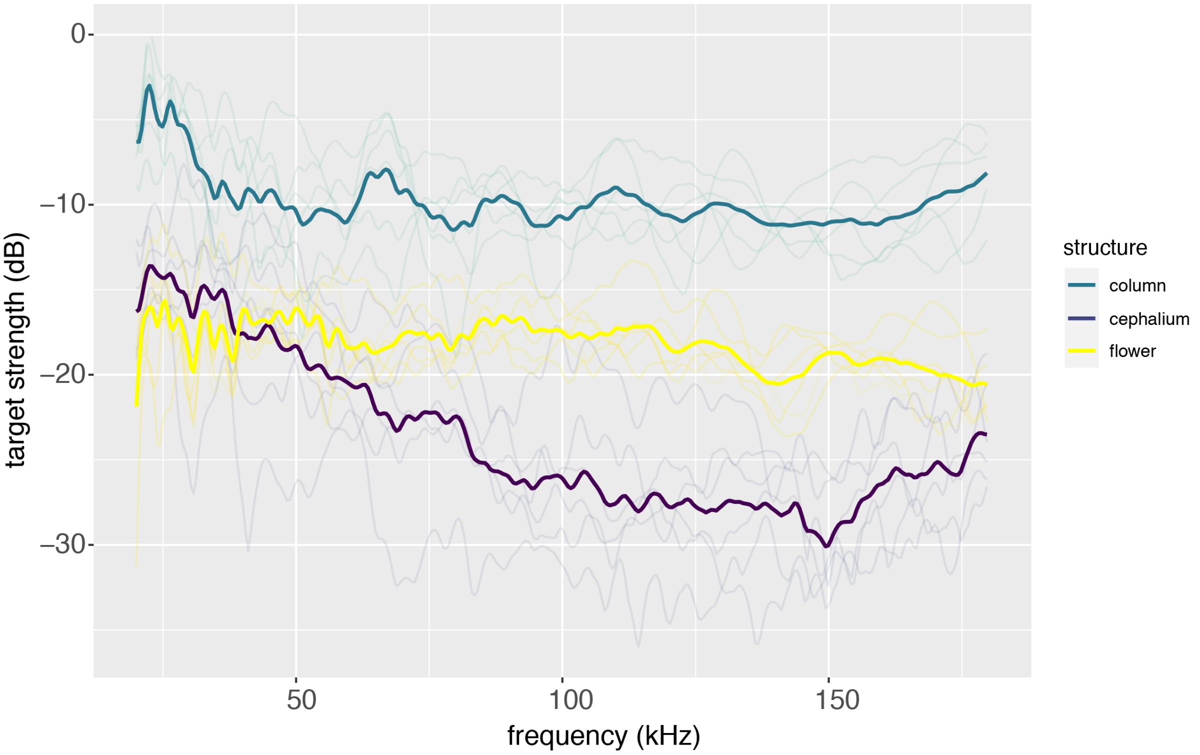

Fig. S2. Averaged echo spectra for the three different cactus structures, column, cephalium and flower. Each single spectrum (thin, transparent lines) represents the average spectrum of 10 measurements from one cactus specimen. The bold lines give the average of 6 specimens.

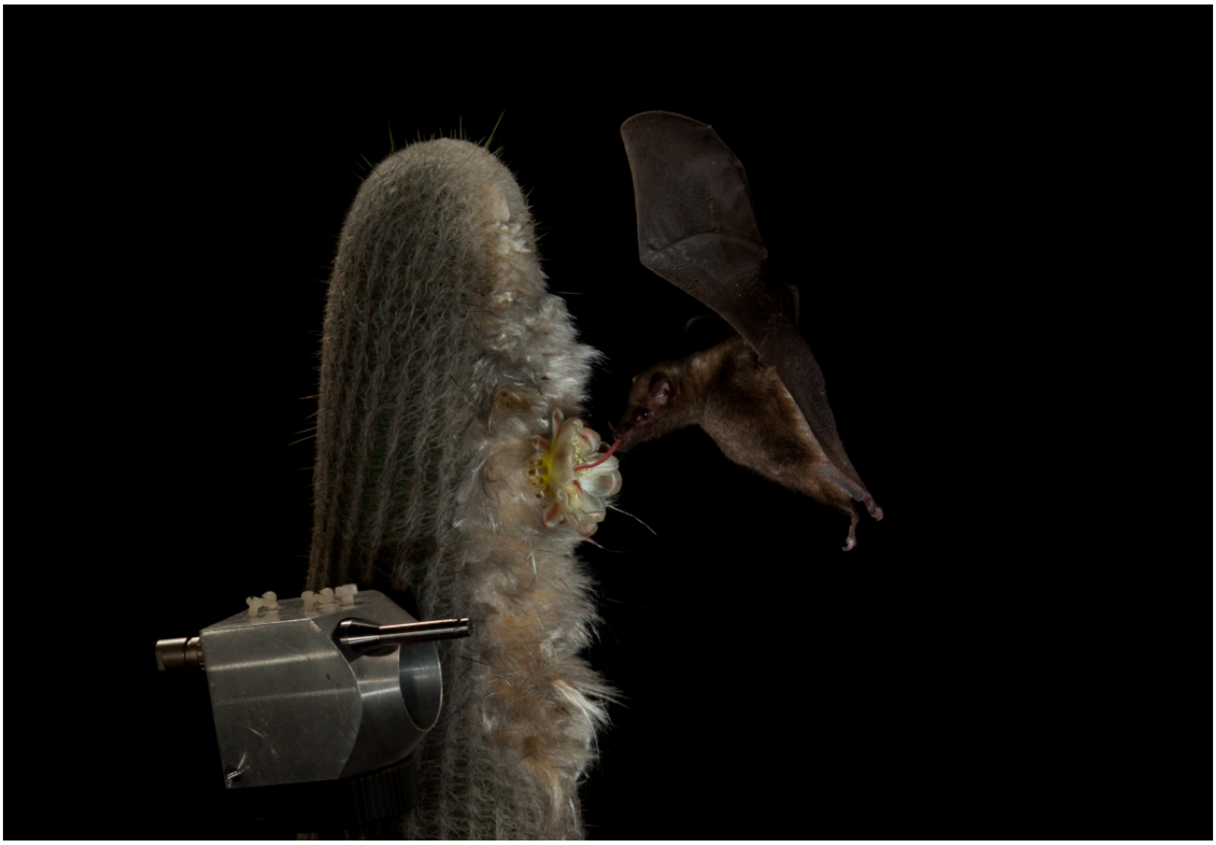

Fig. S3. Geoffroy's tailless bat (*Anoura geoffroyi*) drinking out of a flower of *Espostoa frutescens* while we recorded the echolocation calls during approach. We used a 1/4” G.R.A.S. microphone placed next to the flower (photo credit: ©MerlinTuttle.org).
